# Childhood Disadvantage and Later-Life Multimorbidity: Demonstrating Robustness Under Imperfect Identification

**DOI:** 10.1093/geroni/igab046.1911

**Published:** 2021-12-17

**Authors:** Brayan Seixas, James Macinko

**Affiliations:** UCLA, Los Angeles, California, United States

## Abstract

This study assesses the relationship between indicators of early childhood disadvantage and later-life multimorbidity within a nationally representative sample of Brazilians aged 50 and over (n = 9,412). Data come from the baseline assessment (2015/2016) of the Brazilian Longitudinal Study of Aging (ELSI). We employed survey-weighted Ordinary Least Squares regression to estimate the effects of individual and combined measures of childhood disadvantage on the total number of chronic conditions in later life. Mediation analysis assessed whether adult socioeconomic status (SES) mediated the relationship between childhood disadvantage and chronic conditions. We found that individual and combined measures of childhood disadvantage were associated with the total number of chronic conditions, even after controlling for potential confounders. Mediation analysis suggested that part of the effect of childhood adversity is mediated by higher SES in adulthood (~10%). A formal strategy of sensitivity analysis showed that omitted variable bias is extremely unlikely. To rule out the observed effect, an unobserved hypothetical confounder would need the explanatory power of the residual variance of both the independent and the dependent variables that is at least 30 times larger than that of BMI or 5 times larger than the explanatory power of age. Results should inform efforts to strengthen interventions targeting early childhood development and to improve other key inputs (such as education) to enhance adult SES and lessen the impact of early life stressors on health in older adulthood.

